# Overview of post-discharge predictors for psychiatric re-hospitalisations: a systematic review of the literature

**DOI:** 10.1186/s12888-017-1386-z

**Published:** 2017-06-24

**Authors:** R. Sfetcu, S. Musat, P. Haaramo, M. Ciutan, G. Scintee, C. Vladescu, K. Wahlbeck, H. Katschnig

**Affiliations:** 1 National School of Public Health, Management and Professional Development, Bucharest, Romania; 2 Psychology Department, Spiru Haret University, Bucharest, Romania; 3 National Institute for Health and Welfare, Mental Health Unit, Helsinki, Finland; 4 Victor Babes University of Medicine and Pharmacy, Timisoara, Romania; 5 IMEHPS, Vienna, Austria

**Keywords:** Readmission, Post-discharge factors, Aftercare, Community care, System responsiveness, Social support, Contextual factors, Systematic review

## Abstract

**Background:**

High levels of hospital readmission (rehospitalisation rates) is widely used as indicator of a poor quality of care. This is sometimes also referred to as recidivism or heavy utilization. Previous studies have examined a number of factors likely to influence readmission, although a systematic review of research on post-discharge factors and readmissions has not been conducted so far. The main objective of this review was to identify frequently reported post-discharge factors and their effects on readmission rates.

**Methods:**

Studies on the association between post-discharge variables and readmission after an index discharge with a main psychiatric diagnosis were searched in the bibliographic databases Ovid Medline, PsycINFO, ProQuest Health Management, OpenGrey and Google Scholar. Relevant articles published between January 1990 and June 2014 were included. A systematic approach was used to extract and organize in categories the information about post-discharge factors associated with readmission rates.

**Results:**

Of the 760 articles identified by the initial search, 80 were selected for this review which included a total number of 59 different predictors of psychiatric readmission. Subsequently these were grouped into four categories: 1) individual vulnerability factors, 2) aftercare related factors, 3) community care and service responsiveness, and 4) contextual factors and social support. Individual factors were addressed in 58 papers and were found to be significant in 37 of these, aftercare factors were significant in 30 out of the 45 papers, community care and social support factors were significant in 21 out of 31 papers addressing these while contextual factors and social support were significant in all seven papers which studied them.

**Conclusions:**

This review represents a first attempt at providing an overview of post-discharge factors previously studied in association with readmission. Hence, by mapping out the current research in the area, it highlights the gaps in research and it provides guidance future studies in the area.

**Electronic supplementary material:**

The online version of this article (doi:10.1186/s12888-017-1386-z) contains supplementary material, which is available to authorized users.

## Background

Readmission, rehospitalisation or recidivism are just a few of the terms used interchangeably in the literature to describe repeated episodes of inpatient care, a phenomenon that is often unwelcomed by the patient and costly for the health care system [1]. Readmission rates are a widely used indicator of health care quality, with the underlying assumption being that high readmission rates are related to substandard care [2]. For severe mental disorders the topic of readmissions is relevant due to the high frequency of the event, a study conducted in USA showing that mood disorders and schizophrenia have the highest number of all-cause 30-day hospital readmissions among adult Medicaid patients [3]. Additionally, the need for evidence supporting community mental health services and their role in preventing unplanned hospital readmissions has focused the rehospitalisation research efforts in the post-discharge period [4]. In the last few decades the volume of research on the association of post-discharge factors and rehospitalisation has gradually increased [4], as post-discharge factors have started to be studied as predictors for rehospitalisation [5], distinctively from pre-discharge factors [6]. As a diversity of factors can appear in the post-discharge period, subcategories of post-discharge factors have emerged, in time, such as transitional interventions [3], continuity of care [7] or family interventions [8]. However, the results of studies in this area are often inconsistent, one particular example being the impact of poor access to adequate community-based aftercare on hospital readmission rates.

The need for a systematic review of this evidence has become evident in the context of the Comparative Effectiveness research on Psychiatric HOSpitalisation by record LINKage of large administrative data sets (CEPHOS-LINK)^1^ study, a FP7 funded EU project. The overall objective of the CEPHOS-LINK study was to compare differences in rehospitalisation outcomes for adult patients with a psychiatric diagnosis after an index discharge. Additionally, the project aimed at identifying patient, service and health system factors which affect rehospitalisation patterns by analysing (with record linkage methods) data for large, unselected patient populations contained in administrative health service utilization databases in six European countries. Therefore, the need for a more comprehensive understanding of all the factors impacting on readmission rates has motivated a series of reviews on readmission and the association with four different categories of factors: pre-discharge variables [9], post-discharge variables, system variables [10] and comorbidity [11].

The current review focused on post discharge variables with the aim to identify and categorise previously studied post-discharge factors in relation with readmission rates. In the CEPHOS-LINK project, the results of this systematic investigation have contributed to the theory guided selection of post-discharge variables employed for the record linkage studies. In the wider context of post-discharge variables research, by mapping out the current research in the area, this review highlights the gaps in research and provides guidance for future studies in the area.

## Methods

### Search strategy and screening process

Comprehensive literature searches were conducted in the following electronic bibliographic databases: Ovid Medline, PsycINFO, ProQuest Health Management and OpenGrey. In addition, Google Scholar was utilized. Following the CEPHOS-LINK protocol as well as the PRISMA guidelines research articles focusing on the association between mental health and readmission were searched by using combinations of keywords describing psychiatric disorders and readmission (MeSH terms or free text, depending on the database). The references of all included articles were manually checked for additional studies. The search strategy is presented in detail in the Additional file 1. The resulting reference list was subsequently screened for eligibility by two pairs of independent researchers (RS, LS, VD, EL). Discrepancies were resolved by discussion or by the assessment of a third researcher, until consensus on inclusion of the study was reached.

### Inclusion and exclusion criteria

*Type of studies:* Studies published between January 1990 and June 2014 were included. No restrictions regarding language or publication status were used. Quantitative studies were selected for this systematic review, including both observational and intervention studies. Qualitative studies and case reports were excluded. Papers not including original data, such as editorials, letters to the editor, commentaries, were excluded as well as theses and dissertations and other reviews. To be eligible for this systematic review, the studies had to report data on the association between post-discharge variables and readmission of patients with a main psychiatric diagnosis at discharge. Publications including either bivariate or multivariate analysis were taken into consideration.

### Type of participants

Only studies examining adult populations (age ≥ 18 years) having been discharged from in-patient health care were included in the review.

### Predictors

Post-discharge factors were defined as factors measured at individual level in the time interval between an index discharge and the first readmission. In order to be considered significant, the authors of the original papers had to report a significance level of *p* < .05. The actual discharge process and associated interventions were considered to be pre-discharge variables as well as all factors related to the index inpatient stay. In studies analysing multiple readmissions, post-discharge variables were considered only if measured in the period following the index discharge. The duration of the follow-up period did not represent an exclusion criterion and neither did the type of admission (voluntary vs. involuntary), the type of discharge (e.g. delayed, against medical advice, etc.) or the number of previous/subsequent admissions. General socio-economic variables (e.g. housing situation, income, etc.) were considered post-discharge variables if they were specifically measured in the post-discharge period; these were also excluded if measured at system level rather than individual level. Classical pharmacological studies on how medication prevents relapse/readmission were not included. Transitional interventions starting in the pre-discharge period were also excluded, even if they continued in the post-discharge period.

### Outcomes

Studies not covering the issue of readmission were excluded. Transfers to other services (e.g. general health care, specialized programs, residential care) or admissions to day hospitals or community programs were not included either. All types of readmission indicators have been considered (e.g. readmission rates, survival in the community, time to readmission, etc.).

### Quality assessment

In order to assess the quality of the included papers a tool developed by the CEPHOS-LINK team working on pre-discharge factors was employed [9]. Assessment criteria included in the tool were: representativeness of the target population to the general psychiatric inpatient population; generalizability of the hospital or unit (mainly not diagnostically specialised); participation rate and completeness of follow-up; coverage of hospital readmissions (whether to all available facilities or only to the same hospital of index discharge); controlling for confounding factors in the statistical analyses. Each study was individually assessed by two reviewers [RS, MC]. Disagreements were mediated by a third researcher [SM].

### Data extraction

Available data on variables associated with readmission were independently extracted from the included studies by two researchers [RS, MC]. DistillerSR (Evidence Partners Incorporated, Ottawa, Canada), a Web-based systematic review software^2^ was used for this step, as it allowed us to adopt an iterative approach to coding. Extracted data included the following: aim of the study, the number and category of participants (e.g. veterans), study design, recruitment interval, follow-up interval, main outcome, included diagnostic groups, key factors affecting readmission and their definition. These are presented in the Additional file 2. For papers published in other languages than English, colleagues proficient in the respective languages from the CEPHOS-LINK consortium have been involved in the assessment and data extraction process.

### Data synthesis

A meta-analysis was not conducted due to the high variability in design, population, and the factors investigated by the included studies. For example, in some studies, only schizophrenic patients were included whereas in others the participants are recruited from acute wards. The results are organised into four categories adapted from the framework proposed by Klinkenberg et al. [6], respectively: 1) individual vulnerability (e.g. post-discharge symptoms, behaviours or socio-economic related factors), 2) aftercare factors (e.g. referral to an aftercare agency, follow-up, receipt of psychotherapy, outreach and mobile, day treatment), 3) community care and service responsiveness (e.g. case management, continuity of care), and 4) contextual factors and social support (e.g. community attitudes).

In the results section, factors included for each of these four categories, are presented synthetically in a tabular format. Also, the results of the original articles are summarised, keeping in line with the terminology used by the authors.

## Results

### Results of the search strategy and screening

**Fig. 1:**
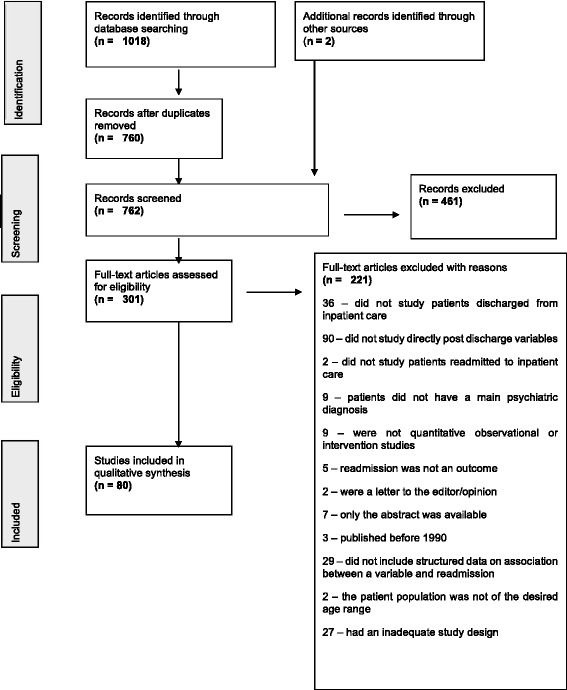
Studies selection flowchart

A total number of 1018 references were retrieved and after duplicates were removed 760 titles remained. 301 full text papers were retrieved, and 2 additional papers were included. Out of these, 221 were subsequently excluded based on exclusion criteria (details are provided in Fig. 1) with a total number of 80 papers being included in the review.

### Studies description

From a geographical distribution point of view, some diversity was observed among the 80 included studies, which reported data from 15 countries, 1 included data from 2 countries and another included data from 4 Nordic countries. The majority of the studies (59/80) were conducted in English speaking countries, with more than 50% of these coming from the USA, almost 15% from Australia, four from UK and two from Canada. None of the studies included low-income countries.

Looking at the design, six of the studies included were intervention studies (out of which five were Randomised Controlled Trials (RCTS)) and 74 were observational. Among the observational studies, 46 were cohort studies (42 prospective and four retrospective), 18 were case-control studies, and four were naturalistic experiments. Ten studies used single large administrative databases [12–21] and other eight were record linkage studies [22–29].

Most follow-up studies (73%) investigated readmission in the first post discharge index year or in a shorter time interval, the rest being distributed between medium term follow-up studies (more than one but less than three years) and follow-up for time intervals of three or more years (19%). The actual follow-up period varied from one month (28/30 days) to 16 years, but in most cases standard follow-up time intervals were used: one month (8%), three months (8%), six months (17%) and 12 months (32%).

In 32 studies, no diagnostic limitation was imposed for inclusion of patients. Schizophrenia and related disorders (coded as F2 ICD-10 category) were studied in 33 studies and mood disorders (F3 ICD-10) in 18 studies. Six studies directly targeted substance use disorders (SUD) and patients with either anxiety or personality and organic disorders were included in other 10 studies. In terms of the dimension of the investigated sample, the included studies greatly varied, with the size of the population ranging from 35 to 128.893 included cases.

### The quality assessment of the papers included

The results of the quality assessment indicated that only seven out of the 80 papers had a high quality (i.e. met five or more of the evaluation criteria), 32 were of poor quality (i.e. met two or less of the evaluation criteria) while the rest had an average level of quality (i.e. met three or four of the evaluation criteria). A more detailed analysis of the number of papers which have met each of the six evaluation criteria is included in Table 1.

**Table 1:** Number and percentage of papers which fulfil the quality criteria

	Representativeness	Participation rate > 90%	Generalizability	Lost to follow-up < 10%	Readmission to all hospitals	Adjustment for confounding factors
Yes	26 (32.5%)	25 (31.2%)	66 (82.5%)	40 (50.0%)	40 (50.0%)	32 (40.0%)
No	51 (63.7%)	37 (46.2%)	12 (15.0%)	15 (18.7%)	35 (43.7%)	44 (55.0%)
Unclear	3 (3.7%)	18 (22.5%)	2 (2.5%)	25 (31.2%)	5 (6.2%)	4 (5.0%)

Most of papers included were not representative of the general psychiatric population discharged from an inpatient service, the patient populations often being composed of selected sub-groups of patients based on criteria such as age, diagnostic or service use patterns (e.g. frequent users). Additionally, only about one third of the studies (31,2%) reported a participation rate over 90% of the selected population and only 50,0% of the papers clearly reported a percentage of patients lost at follow-up lower than 10%. One reason for this situation is that in 22,5% for the first and 31,2% for the second, the fulfilment of these criteria could not be decided based on the reported data. However, a big percentage of the papers (82,5%) reported on data collected from general psychiatric hospitals or inpatient psychiatric units in a general hospital. In around half of the papers the analysis considered readmission to all possible hospitals. Most of the studies used multivariate analytical methods, i.e., confounders were controlled for, but only around 40% clearly reported adjusting for clinical of demographical pre-discharge factors which play an important role on readmission.

### Post-discharge factors impacting on readmission

In total, 59 different factors were identified and distributed into four categories: individual vulnerability, aftercare factors, community care and service responsiveness and contextual factors and social support. The results from individual studies for each of these categories are summarised and discussed in the following sections.

### Individual vulnerability factors

Post-discharge factors related to the individual vulnerability were reported in 39 papers, among those most frequently studied being compliance to treatment [17, 30–37] or to follow-up appointments [31, 33, 36, 38–40], the type of housing the patient was discharged to [14, 24, 30, 34, 39–45], and the post-discharge alcohol/substance abuse [24, 30, 34–36, 40, 46, 47]. The significant results as well as the type of analysis in which these factors were found to be significant (bivariate vs multivariate) as well as the other variables that authors adjusted their results for are included in Table 2.

**Table 2:** Synthesis of the main bivariate and multivariate significant results regarding individual vulnerability factors

Individual vulnerability	No. of sig. Studies/Total no. of studies	Main significant results bivariate	Main significant results multivariate
Compliance (compliance/noncompliance to treatment, compliance/noncompliance to appointments)	12/16 Protective factor	Protective factor: 7	Protective factor: 6
Housing and living arrangements (own home vs rest; homelessness, staffed vs non-staffed group homes; family of origin vs alone or family of procreation)	7/12 Mixed results	Protective factor: 1 Discharged to own home vs boarding home Risk factor: 1 Staffed vs non-staffed group homes	Risk factor: 5 Homelessness. Living in the family of origin as compared to the family of procreation or living alone. Living alone as compared to living with a parent or relative or in supported housing. Patients living in nursing homes vs all other. Living with other people vs living alone
Symptoms related (alcohol/substance abuse, unavoidable acute relapse in the course of a chronic condition)	6/10 Risk factor	Risk factor: 3 Alcohol abuse. Substance use disorder post-dscharge vs pre-discharge. SUD diagnosis at follow-up car	Risk factor: 3 [Substance abuse/dependence. Alcohol abuse: risk factor. Drug misuse
Post-discharge behaviour (self-harm, behavioural problems, violence, homicide/suicide, abnormal behaviour)	3/5 Risk factor	Risk factor: 1 Behav. Problems (e.g. violence, police involved, homicide/suicide	Risk factor: 2 Self-harming post. Not grooming
Financial factors (receipt of benefits, employment)	4/8 Mixed results	Risk factor: 2 Being unemployed after discharge, receipt of benefits. Receipt of DSP, unemployment	Protective factor: 1 Regular job vs occupational therapy or unemployment. Risk factor:1 Being on benefits
General well-being in the period post discharge (psychosocial stress, quality of life, life events)	2/3 Risk factor	Risk factor: 2 Satisfaction with treatment. Dissatisfaction with family	Risk factor: 1 Dissatisfaction with family

Psychiatric medication adherence and compliance with follow-up appointments were found to be significant predictors of readmission in 12 out of 16 papers, being some of the most researched and confirmed individual vulnerability factors. The type of housing the patients were discharged to was the second most researched individual factor, 7 out of 12 papers founding a significant association with the readmission rates, with patients being discharged to their own home having better outcomes. The negative impact of alcohol/substance abuse comorbidity was studied in ten papers, but was only confirmed in six of these as a risk factor for readmission. A series of individual factors related to financial aspects, general well-being in the period post discharge as well as post-discharge behaviour have been also studied, albeit in a small number of papers each and with mixed or inconclusive results. In summary, for housing and financial factors, the results were mixed with respect to their predictive capacity of readmission risk while for most of the other factors they were inconclusive due to the reduced number of conducted studies and varying quality.

### Aftercare related factors

In previous studies the receipt of aftercare was defined as “following through on treatment recommendations for aftercare” and included either a single contact with an aftercare agency after hospital discharge, a visit to the psychiatric emergency room, or a certain number of clinic visits within a specific period of time after discharge [48]. In our study we have expanded this category to also include the referral to an aftercare agency (e.g. Community Mental Health Centre, a structured aftercare programme), follow-up (or lack of follow-up) by different categories of health professionals (e.g. general practitioners (GPs), psychiatrists, nurses) or means (e.g. by telephone, home visits) and within different post-discharge time intervals (e.g. 7 days, 30 days) as well as other types of service use episodes (e.g. receipt of psychotherapy, outreach and mobile, day treatment). Below we grouped the results based on the type of agency that provided the aftercare. We also took into consideration the issues of follow-up time interval and the intensity of aftercare, as both of these can have a moderating effect on the effectiveness of aftercare in reducing readmission rates. Altogether seven different subcategories have emerged: follow-up in primary care, referral to outpatient services, type of provider and locus of care, post-discharge access to treatment (medication prescription), psychiatric follow-up within seven days from discharge, psychiatric follow-up within 30 days from discharge, long term psychiatric follow-up, follow-up in day treatment settings. In Table 3 the significant bivariate and multivariate results are summarized and information about the variables controlled for is included for all aftercare related factors. A narrative summary of main results is also provided for each aftercare subcategory.

**Table 3:** Synthesis of the main bivariate and multivariate significant results regarding aftercare factors

Aftercare factors	No. of sig. Studies/Total no. of studies	Main significant results bivariate	Main significant results multivariate
Follow-up in primary care	7/8 Mixed results	Protective factor: 3 Discharge plan being sent to GP. Receiving home aftercare. Contact cu GP PD	Protective factor:3 Discharge plan being sent to GP. Discharge plan being sent to GP. Receiving home aftercare
		Risk factor: 2 Being registered with a PCU. Receiving more family physician hours	
Referral to outpatient services, type of provider and locus of care	4/7 Mixed results	Protective factor: 1 Community. Psychosocial Care Center	Risk factor: 3 Services being provided by the local AMHT. Referral to aftercare. Being seen by a psychiatrist during the first aftercare appointment
Post-discharge access to treatment (medication prescription)	3/4 Risk factor	Risk factor: 3 Having a prescription medication fill in the week following discharge. Receiving subsidized or free medication *Receiving medication for more months	No significant results
Follow-up within seven days from discharge	4/5 Mixed results	Risk factor: 2 Follow-up by the AMHT within 7 days. Contact in the community on the day of discharge.	Risk factor: 2 Follow-up by the AMHT within 7 days. Contact in the community on the day of discharge.
		Protective factor: 1 24-h follow-up	Protective factor: 1 OP treatment from CMHC team within 7 days
Follow-up within 30 days from discharge	6/6 Mixed results	Risk factor: 1 Having 30 days follow-	Risk factor: 1 Having 30 days follow-up (NAdj.)
		Protective factor: 2 *Having two or more sessions of outpatient mental health care. OP visits	Protective factor: 5 Attending one post discharge appointment *Having two or more sessions of OP mental health care. OP visits. OP mental health care. *Receiving substance use disorders treatment
Long term follow-up	4/10 Protective factor	No significant results	Protective factor: 4 Visiting a mental health clinic after discharge. *Receiving aftercare. *Receiving intense monitoring in the PD period
Day treatment	2/4 Mixed results	Protective factor: 1 Receiving day care as a structured program	No significant results
		Risk factor: 1 Receiving psychiatric day care	

*indicates that the results are significant only for subgrups of the studied population

* indicates that the results are significant only for subgrups of the studied population

### Follow-up in primary care

Planning and following through post-discharge aftercare in primary care, by a social worker or nurse was studied in eight papers and found to be significant in seven of these, with mixed results. Two papers showed that sending the discharge plan to the GP for follow-up is effective in reducing the readmission risk within 28 days after the index discharge when compared with referral to acute mental health services [49, 50], while another found that the actual contact with the GP is also reducing risk of being readmitted to hospital [51]. However, more GP treatment time was found to increase the risk for rehospitalisation in one study [52], while just being registered with a primary care unit did not make a significant difference in another [33]. In a randomized controlled study, Sharifi et al. found that when a GP and a social worker made home visits once during the month after discharge from the hospital wherein they provided education and treatment (home aftercare), it led to a reduction in rehospitalisation rate [53]. Similar results were obtained when the home visits were conducted by psychiatric nurses only [54, 55]. In summary, planning for and having direct contact with a primary care provider in the post-discharge period can reduce readmission rates but just being registered with a GP makes no difference and as the intensity of the contact increases it may actually lead to an increased readmission risk.

### Psychiatric aftercare

Monitoring follow-up after psychiatric hospitalization within seven and 30 days of discharge are routinely used healthcare effectiveness measures. These are defined as the percentage of discharged patients who had an outpatient visit, an intensive outpatient service, or partial hospitalization with a mental health provider within seven or within 30 days of discharge (National Committee for Quality Assurance Healthcare Effectiveness and Data Information Set [HEDIS])^3^. However, some of the included studies have utilized longer follow-up time intervals (e.g. 180 days, one year), which is why we have reported the results under separate subsections based on the follow-up interval for psychiatric aftercare.

### Referral to outpatient services, type of provider and locus of care

Referral to outpatient services was studied by seven papers and was found to be significant in four of these. Having a referral to a psychiatric aftercare program (e.g. outpatient care, foster care, or a group home) significantly increased the risk of rehospitalisation within six months of discharge [56] as well as the aftercare provider being a psychiatrist vs. a non-psychiatrist [57] but the setting where the care was provided (locus of care) had no significant effect [58]. The use/lack of use of the Community Mental Health Centre (CMHC) as regular source of care was equally found to have no effect by one study [30]. Two other studies reported contradictory results, with one arguing that being referred to community psychosocial support units lowered the odds of multiple readmissions when compared to those referred to usual outpatient care [33] and the second showing that patients for whom follow up after discharge was planned at the local adult mental health service were more likely to have an earlier readmission than those who were referred back to their GP or other service providers for follow up [52]. In summary, the results for post-discharge referral to outpatient services were mixed, with three papers having found it to be a risk factor and one a protective one.

### Post-discharge access to treatment

In total seven studies have addressed the post-discharge access to pharmacological and psychological treatment provided in the post-discharge period. Out of these, four studies reported on the relationship between medication in the post-discharge period and readmission. The results indicate that patients who received subsidized or free medication^4^ were more likely than those who did not receive it to have multiple readmissions [33]. However, the duration of medication receipt also played a role, as those who were not hospitalized reported receiving medication^5^ for significantly fewer months than schizophrenic patients who were hospitalized [59]. When measuring the receipt of medication as the percentage of patients receiving a prescription fill for a mental/substance use disorder (M/SUD), the results were similar: more medication leads to more readmissions [20]. However, a change in medication within the last month had no distinctive impact on readmissions [36]. Receipt of psychotherapy was included in three studies, but was not significant in any of them [33, 58, 59]. In summary, more medication in the post-discharge period is a risk factor but receipt of psychotherapy has no impact on readmission rates.

### Follow-up within seven days from discharge

In total, five studies have analysed the impact of follow-up in the first seven days after discharge on readmission rates, with mixed results. One study analysing the impact of follow-up on the day of the discharge proved that having a contact in the community on the day of discharge (24 h follow-up) is effective in reducing readmission rates [60], and so is receiving outpatient treatment at a CMHC within the first seven days of discharge [20]. By contrast, a study by Pfeiffer and al. [61] reported that follow-up within seven days did not determine a reduction in readmission after discharge, while two other provided evidence that a contact in the community on the day of discharge [52] and follow up by the mental health team within seven days of discharge lead to increased readmission [50].

### Follow-up within 30 days from discharge

Six studies have tested the impact of follow-up within 30 days from discharge on readmission rates. In this case, the results reported were more consistent as compared to studies on follow-up within seven days from discharge. In five studies, for more contact significantly lower readmission rates were observed [13, 18, 21, 27, 62]. This association seemed to be stronger among middle-aged and older patients than it was among younger patients [21]. However, a study of voluntary readmission on schizophrenic patients showed that the receipt of follow-up services from a community mental health centre within 30 days increases the readmission risk [12].

### Long term follow-up

Among the ten studies that studies longer term follow-up, only one found clear evidence that outpatient visits within 180 days of index discharge can reduce readmission rates [23]. Three others found long term follow-up to be effective only for subgroups of patients [19, 25, 63]. For example, increased monitoring led to decreased rehospitalisation among depressed patients with a comorbid substance use disorder in one study [19] and, another study following patients for one year has found an increased readmission risk for psychotic patients without aftercare [64].

In terms of aftercare intensity, the number of visits for medication prescription only or the number of mental health care visits of any type during a six month follow-up did not influence the readmission outcome for patients diagnosed with a psychotic illness [59], neither did the number of contacts with mental health providers [63] and four others could not prove that the extent of subsequent patient mental health is a valid predictor for readmission [25, 28, 34, 58].

### Day treatment

Day treatment service use was included in four studies, of which only two could establish a significant relation with readmission in bivariate analysis. A case-control study of rapid readmission shows that fewer rapidly readmitted psychiatric inpatients are discharged to a structured program (e.g. day hospital) as compared to matched samples of patients with long community tenure or without any readmission. Interestingly enough, the utilization of the day-care unit at the public health centre and workshops in the community was positively correlated with rehospitalisation but this effect did not remain significant in multivariate analysis [37]. The use of post-discharge day treatment services by older patients hospitalized for depression had no effect on readmission rates [31], and neither did the number of days spent in day-care by patients with schizophrenia and related disorders [28]. In summary, the evidence we found for the impact of day treatment on readmission rates is mixed, and of poor quality.

### Community care and service responsiveness

The significant bivariate and multivariate results for community care and responsiveness are summarized in Table 4 and a brief narrative summary of main results is also provided.

**Table 4:** Synthesis of the main bivariate and multivariate significant results regarding community care and service responsiveness factors

Community care and service responsiveness factors	No. of sig. Studies/Total no. of studies	Main significant results bivariate	Main significant results multivariate
Case management programs	7/12 Mixed results	Risk factor: 5 Receiving or requiring more intensive case management. Being assigned to ACT team. More outreach care. More case management. Having intensive case management outreach	Risk factor: 1 Assignment to a residential program and/or to case management
			Protective factor: 1 Case management
		Protective factor: 1 Case management	
Compulsory outpatient treatment	5/5 Mixed results	Protective factor: 2 Being on CTO. Being on CTO	Risk factor: 2 Being on CTO at discharge
			Protective factor: 2 Community initiated CTOs. CTO + intensive community care
Continuity of care practices and programs	9/14 Mixed results	Protective factor: 7 Being part of a CoC research program. *Receipt of continuous treatment. Being followed-up by inpatient staff in a hospital setting. *Being followed-up continuously. Receiving a complex PD intervention from the OP psychiatrist. Follow-up through decision support tool. Follow-up through mobile app	Risk factor: 1 Service connectedness
			Protective factor: 1 Reviewing the individual service plan, a change in the treating team

*indicates that the results are significant only for subgrups of the studied population

* indicates that the results are significant only for subgrups of the studied population 

### Case management programs

Case management programs or adaptations of it were studied in twelve studies [30, 41, 65–73]. In five of these, case management was found to have no effect on readmission, in two studies it increased readmission, while in the remaining five it decreased readmission. However, the overall quality of the papers included was rather poor with only two studies (with mixed results) having their findings confirmed in multivariate analysis.

### Compulsory outpatient treatment

Another five studies investigated the effectiveness of Community Treatment Orders (CTO) in reducing readmission rates [74–77]. In four of these, results indicated a potential positive effect of CTO on readmission rates, but due to the heterogeneity of studies it is difficult to draw unequivocal conclusions. As this topic has been extensively discussed elsewhere [78], we will not further explore it here.

### Continuity of care practices and programs

Continuity of care was one of the variables of interest for seven individual studies, out of which one focused solely on the continuity of the treatment. While the definitions of continuity of care varied from study to study, they all included at least one of the three types of continuity: informational continuity, management continuity or relational continuity, as described by Haggerty et al. [79].

Three of these studies showed that continuity of care contributed to reduced readmissions (with two showing partial support), three showed no support for this relationship, and one found evidence that increased continuity of care led to an increased risk of readmission.

Follow-up in the ward, by the same staff, significantly reduced the number and length of hospitalizations as compared to the traditional system of follow-up in an outpatient clinic [80]. In one study continuity of treatment made no difference for affective disorders patients but it protected schizophrenic patients from rehospitalisation [59] while in another intervention study even if a reduction in total readmissions could not be proved to be significant, a decrease of involuntary readmissions was observed [81].

Three studies looking at collaboration between hospital and community services [60], at the continuity of care^7^ for patients with both substance abuse and major psychiatric disorders [27] and at different levels of continuity of care^8^ for patients with schizophrenia and related disorders [28] found no significant effects.

Finally, service connectedness was found to increase the readmission risk of patients with severe mental illness by another study [24].

Other seven studies have analysed the effectiveness of specific programs or interventions in reducing readmission rates through continuous care. Out of these, four studies analysed three different relapse prevention programs and all were found effective in reducing the readmissions. Two of the programs (a decision support tool and a mobile app) were designed for patients with schizophrenia [82, 83], and one (Triggers Intervention and Prevention System) for frequent users of inpatient services [84, 85]. Other effective interventions included: reviewing the individual service plan [86] and a specific interventions addressing medication education, symptom education, service continuity, social skills, daily living, daily structure, and family issues [87]. Conducting a full intake interview at aftercare visit [57] had no significant effect. Finally, a study aiming to assess whether (and in what way) research procedures may affect outcomes [88] found that just being part of a research program resulted in a reduced readmission rate of 31% in the experimental group^9^ vs. 51% in the control group.

In summary, nine of the 14 studies addressing continuity of care practices or specific intervention were significant. However, for the only two studies that have conducted multivariate analysis the results are mixed.

### Contextual factors and social support

The significant bivariate and multivariate results for contextual factors and social support are summarized in Table 5 and a brief narrative summary of main results is also provided.

**Table 5:** Synthesis of the main bivariate and multivariate significant results regarding contextual factors and social support factors

Contextual factors and social support	No. of sig. Studies/Total no. of studies	Main significant results bivariate	Main significant results multivariate
Geographical variables (proximity to services)	2/2 Risk factor	Risk factor: 1 Proximity to hospital	Risk factor: 1 Being discharged to a location near a Narcotics Anonymous meeting place and in an area with low educational attainment
Support/lack of support of the family (criticism, maladaptive functioning, stigma)	4/4 Protective factor	Protective factor:1 Supportive comments	Risk factors: 3 Maladaptive family system functioning. Criticism or rejection of the patient. Family’s agreement with hospitalization
Peer support	1/1 Protective factor	Protective factor: 1 Being assigned to a recovery mentor	No significant results

### Geographical variables

Several geographical variables were included in a study aiming to analyse neighbourhood and individual factors predicting rehospitalisation within one year among patients who were dually diagnosed with at least one mental disorder and a substance use disorder [89]. The results showed an increased likelihood of being readmitted for two of these variables, i.e. the patient being discharged after hospitalization to a location near a Narcotics Anonymous meeting place, and living in an area with low educational attainment. Another study found that individuals who lived in the same city as the hospital had a higher likelihood of readmission than those who lived in the greater metropolitan area [33].

### Support of the family

The role of the presence or absence of family support in readmission was studied by four independent studies. Family’s stigma^10^ was found to increase the one year readmissions of individuals with bipolar and psychotic disorder in need of hospitalization [90], and maladaptive family system functioning^11^ was the strongest independent predictor of geropsychiatric rehospitalisation [43]. Also, criticism from family was found to be associated with greater risk for rehospitalisation [30]. At the same time, a familial supportive comment toward the patient decreased the rehospitalisation risk [37].

### Peer support

Peer support has also proved to be effective in reducing recurrent psychiatric hospitalization of individuals with severe mental disorder, with patients who were assigned a peer mentor having significantly fewer rehospitalisation episodes [91].

## Discussion

The purpose of this review was to identify the types of post-discharge variables that may have an impact on readmission rates for patients with a main psychiatric diagnosis. Four categories of post-discharge factors were proposed: individual factors, aftercare, community care and system responsiveness and contextual factors and social support. However, these are not homogenous categories, each of the four including a diverse range of factors as measured by an even more diverse set of indicators.

While it is difficult to separate pre-discharge from post-discharge *individual factors*, a number of authors have succeeded in measuring post-discharge factors in the included studies. Such factors are compliance to treatment and appointments, housing arrangements in the post-discharge period, post-discharge symptom related factors, post-discharge behaviour, the post-discharge financial and occupational situation, as well as the general well-being in the post discharge period. From our results, it seems that compliance is protective for rehospitalisation while post-discharge symptoms related factors, challenging behaviours and a dissatisfaction with the living situation are risk factors for readmission. For housing and financial and occupational status we have found mixed results. Our mixed results for the housing in the post-discharge period are consistent with results found for the pre-discharge period by another CEPHOS-LINK review [9]. However, due to the low quality of the evidence as well as to the great heterogeneity of the papers it is difficult to clearly establish a clear association between the above described factors and the readmission rate.

In the category *post-discharge aftercare related factors,* eight different sub-categories have been identified, with different results for each of these. For follow-up in the primary care we have found mixed results, planning for and having direct contact with a primary care provider in the post-discharge period seems to be effective in reducing readmission rates but just being registered with a GP makes no difference and more intense contact can lead to an increased readmission risk. One interpretation of these results could be that referral to GP may reflect a clinical assessment of lower risk or severity as compared with patients referred to acute services. Referral to more specialized services (e.g. psychiatrist vs. other mental health professional, community mental health teams vs. outpatient follow-up) also seems to increase the readmission risk as does receiving more medication in the post-discharge period. For psychiatric follow-up in the first seven days after discharge we have found mixed evidence while follow-up within 30 days seems to play rather a protective role. Longer term psychiatric follow-up was also partially found to be protective for readmission, although in a few number of studies only. For day treatment the results were also mixed. Presumably, when patients attending these facilities deteriorate clinically, staff may advise them to attend an outpatient clinic or to refer them to hospital, potentially leading to rehospitalisation. Even if these results cannot be used as such to argue about the appropriate level of aftercare as well as the most indicated providers, it provides insight into which aftercare services and providers are more effective in keeping patients outside the hospital. However, these results must be used cautiously as better quality and systematic research is needed in order to draw definite conclusion on the association of the above described aftercare factors and readmission rates.

In terms of *community care and system responsiveness factors*, case management programs seem to be the most common approach used by mental health organizations in order to help clients with severe and persistent mental illness navigate the complex and fragmented healthcare service system. Case management programs or adaptations of it were studied in twelve of the studies reviewed, five of these showing no effect. In two studies it increased readmission rates, while in the remaining four showed a decreased in readmission rates. However, these mixed results tend to offer more support for programs adapted to target patient subgroups than for the classic case management model. For compulsory outpatient treatment our review has found mixed evidence, although a recent systematic review shows that CTOs have no impact on compulsory admissions [92]. An encouraging result of our study is represented by the evidence that specific programs or interventions focusing on reducing readmission rates, whatever their focus might be (i.e. relapse rate, education, skill training). While this result opens the possibility that specific system level interventions can be effective in improving community survival rates, a more in-depth analysis of this topic is needed. Although the topic of continuity of care has been extensively researched [7, 93] results are still inconclusive and primary research focusing on multidimensional measures of continuity of care is needed to better understand the mechanisms at play.

In terms of *contextual factors and social support* post discharge, the research is scarce with only a few papers including these types of post-discharge factors. Among these, the role of the presence or absence of family support in readmission was the most frequently investigated in the reviewed studies and the results consistently showed that readmission can be prevented by working with families, results similar to those found by Pitschel-Walz et al. in their review [8].

### Strengths and limitations

This review had a number of limitations. Since the area of post discharge factors research is both all-encompassing and unstructured in terms of naming conventions, a broad search strategy has been developed and employed. As a result, relevant papers for the topic of post-discharge factors associated with readmissions but focussing on a particular category of factors may have been missed in the search process. For example, the field of compulsory outpatient treatment is well developed, in the recent years several RCTs and systematic reviews having been conducted on this particular topic [92, 94]. Despite this, only five papers have been identified and included in our review. This may be due to the fact that no key term for compulsory treatment was included in the search strategy. The situation is similar for most of the categories of post-discharge factors included in our review and for which the available research extends greatly beyond our reach (e.g. continuity of care, case management). Therefore, we recommend that our results are used rather as a map of post-discharge factors associated with readmission than as actual proof of effectiveness of all the factors analysed and that more focused reviews are employed for effectiveness data regarding particular post-discharge factors.

Another challenge we faced while conducting this review is related to the inconsistent terminology used in the area of post-discharge research (e.g. terms such as aftercare, follow-up, continuous care are poorly defined) as well as to the unstandardized measurement of the same factor across papers (e.g. for follow up within 30 days authors have used, among others: attending one post discharge appointment, having two or more sessions of OP mental health care, OP visits, OP mental health care. This make it difficult to interpret result in a meaningful manner, since what seems to be the same factor measured differently might in fact be just two separate factors. Additionally, very few studies actually include in their studies a temporal dimension, which adds to the complexity of the task of clearly defining individual post-discharge factor. Future studies should address this complexity by employing more focused designs, by embedding a temporal dimension in the research and by operationalizing more clearly variables analysed.

An area for which results are mixed and needs more research is the role of aftercare in the post-discharge intervals of seven and 30 days. While these seem to be the most vulnerable time intervals for readmission, research on the effectiveness of aftercare is still inconclusive, and more studies are needed.

Overall, the literature on post-discharge predictors of readmission must be viewed with caution as studies often reach contradictory conclusions, presumably for many reasons including among others: divergent service characteristics, different populations being examined, differing admission policies and because of methodological and theoretical differences in study design [49].

Finally, the inclusion of both bivariate and multivariate analysis is another limitation of this study. However, taking into consideration the primary purpose of providing an overview of post-discharge factors studied in relation with readmission rates, this approach was preferred in our review.

## Conclusion

Research in the area of post-discharge variables and their impact on readmission rates is unequally developed, with some categories of factors being more extensively researched (e.g. compulsory treatment, continuity of care, case management) while others are still insufficiently addressed (e.g. contextual factors and social support). Even in cases where more research is available, due to high complexity and inter-relatedness of the topic it is difficult to derive definitive conclusions regarding the impact different factors have on readmission rates. Further analyses, including more focused meta-regression studies, are needed to tailor more effective, subgroup specific post-discharge services for persons with a main psychiatric diagnostic.

### Endnotes

1. http://cordis.europa.eu/project/rcn/185457_en.html
2. https://distillercer.com
3. http://www.ncqa.org/hedis-quality-measurement
4. That is, distributed by the government or a health service provider
5. Number of months receiving medication during a 6-month period
6. The ACT program incorporates a broad spectrum of services to patients who have chronic psychiatric conditions and who are especially prone to relapse. This is achieved by active and co-ordinated case management and intensive psychiatric follow-up. The program offers home-based treatment and support to clients and their families. It facilitates the integration of clients into supportive community based networks
7. Measured as the percentage of patients receiving aftercare from the same staff that provided inpatient care
8. Measured as the total number of breaks in the continuity of care in the follow-up period (a break is defined as an episode without any mental health care contacts of at least 90 days) and the total number of days of all breaks in the follow-up period
9. Experimental group patients were intensively assessed on index admission by way of interviews with patients and relatives, and 4 six monthly home visits by psychiatric nurses.
10. Measured as “family’s agreement with permanent hospitalization”
11. Rating of family or social system functioning (%) Effective if not taxed, Chronically ineffective, Maladaptive, Absent or alienated, No longer effective

## Additional files

Additional file 1: Detailed search strategies (includes the specific search strategies used to identify relevant studies for the CEPHOS-LINK systematic review on post-discharge factors and psychiatric readmission) (PDF 82 kb)

Additional file 2: Evaluation table (study description table which includes: author, year and country of each publication, the diagnostic category for the study population, the study design, the aim of the study, the recruitment and follow up interval, the post discharge factors investigated by the study, the pre-discharge factors investigated and whether the factor was found to be significant) (PDF 200 kb)

## Abbreviations

AMHS: Adult Mental Health Service
CMHC: Community Mental Health Centre
CV: Cristian Vladescu
EL: Eva Lassemo
EU: European Union
FP7: Framework Program 7
GS: Gabriela Scintee
HK: Heinz Katschnig
KW: Kristian Wahlbeck
LS: Liljana Sprah
MC: Marius Ciutan
OP: Outpatient
PD: Post-discharge
PH: Peija Haaramo
RS: Raluca Sfectu
SM: Simona Musat
VD: Valeria Donisi

## References

[1] Lien L (2002). Are readmission rates influenced by how psychiatric services are organized?. Nord J Psychiatry.

[2] Fischer C, Anema HA, Klazinga NS (2012). The validity of indicators for assessing quality of care: a review of the European literature on hospital readmission rate. Eur J Pub Health.

[3] Vigod SN (2013). Transitional interventions to reduce early psychiatric readmissions in adults: systematic review. Br J Psychiatry J Ment Sci.

[4] Dausey DJ, Rosenheck RA, Lehman AF (2002). Preadmission care as a new mental health performance indicator. Psychiatr Serv Wash DC.

[5] Krumholz HM (2013). Post-hospital syndrome — an acquired, transient condition of generalized risk. N Engl J Med.

[6] Klinkenberg WD, Calsyn RJ (1996). Predictors of receipt of aftercare and recidivism among persons with severe mental illness: a review. Psychiatr Serv.

[7] Puntis S, Rugkåsa J, Forrest A, Mitchell A, Burns T (2015). Associations between continuity of care and patient outcomes in mental health care: a systematic review. Psychiatr. Serv. Wash. DC.

[8] Pitschel-Walz G, Leucht S, Bauml J, Kissling W, Engel RR (2001). The effect of family interventions on relapse and rehospitalization in schizophrenia--a meta-analysis. Schizophr Bull.

[9] V. Donisi, F. Tedeschi, K. Wahlbeck, P. Haaramo, and F. Amaddeo, “Pre-discharge factors predicting readmissions of psychiatric patients: a systematic review of the literature,” *BMC Psychiatry*, vol. 16, no. 1, p. 449, 2016. 10.1186/s12888-016-1114-0 PMC5162092 27986079

[10] J. Kalseth, E. Lassemo, K. Wahlbeck, P. Haaramo, and J. Magnussen, “Psychiatric readmissions and their association with environmental and health system characteristics: a systematic review of the literature,” *BMC Psychiatry*, vol. 16, no. 1, p. 376, 2016. 10.1186/s12888-016-1099-8 PMC5100223 27821155

[11] L. Šprah, M. Z. Dernovšek, K. Wahlbeck, and P. Haaramo, “Psychiatric readmissions and their association with physical comorbidity: a systematic literature review,” *BMC Psychiatry*, vol. 17, no. 1, p. 2, 2017. 10.1186/s12888-016-1172-3 PMC5210297 28049441

[12] Becker EA, Shafer A (2007). Voluntary readmission among schizophrenic patients in the Texas state psychiatric hospital system. Tex Med.

[13] Bernet AC (2013). Predictors of psychiatric readmission among veterans at high risk of suicide: the impact of post-discharge aftercare. Arch Psychiatr Nurs.

[14] Browne G, Courtney M, Meehan T (2004). Type of housing predicts rate of readmission to hospital but not length of stay in people with schizophrenia on the gold coast in Queensland. Aust Health Rev.

[15] Burgess P, Bindman J, Leese M, Henderson C, Szmukler G (2006). Do community treatment orders for mental illness reduce readmission to hospital?: an epidemiological study. Soc Psychiatry Psychiatr Epidemiol.

[16] Claassen CA, Michael Kashner T, Gilfillan SK, Larkin GL, John Rush A (2005). Psychiatric emergency service use after implementation of managed care in a public mental health system. Psychiatr Serv.

[17] Hassan M, Lage MJ (2009). Risk of rehospitalization among bipolar disorder patients who are nonadherent to antipsychotic therapy after hospital discharge. Am J Health Syst Pharm.

[18] M. Ilgen, K. Hu, R. Moos, and J. McKellar, “Continuing care utilization and readmission to inpatient psychiatric treatment in patients with co-occurring psychiatric and substance use disorders,” *Psychiatr. Serv.*, vol. 59, no. Journal Article, pp. 982–988, 2008. 10.1176/ps.2008.59.9.982 18757590

[19] H. M. Kim, “Intensity of Outpatient Monitoring After Discharge and Psychiatric Rehospitalization of Veterans With Depression,” *Psychiatr. Serv.*, vol. 62, no. 11, Nov. 2011. 10.1176/ps.62.11.pss6211_1346 22211215

[20] Mark T, Tomic KS, Kowlessar N, Chu BC, Vandivort-Warren R, Smith S (2013). Hospital readmission among Medicaid patients with an index hospitalization for mental and/or substance use disorder. J Behav Health Serv Res.

[21] Moos RH, Mertens JR, Brennan PL (1995). Program characteristics and readmission among older substance abuse patients: comparisons with middle-aged and younger patients. J Ment Health Adm.

[22] Bodén R, Brandt L, Kieler H, Andersen M, Reutfors J (2011). Early non-adherence to medication and other risk factors for rehospitalization in schizophrenia and schizoaffective disorder. Schizophr Res.

[23] Grinshpoon A, Lerner Y, Hornik-Lurie T, Zilber N, Ponizovsky AM (2011). Post-discharge contact with mental health clinics and psychiatric readmission: a 6-month follow-up study. Isr J Psychiatry Relat Sci.

[24] Irmiter C, McCarthy JF, Barry KL, Soliman S, Blow FC (2007). Reinstitutionalization following psychiatric discharge among VA patients with serious mental illness: a national longitudinal study. Psychiatr. Q..

[25] Moos RH, Brennan PL, Mertens JR (1994). Diagnostic subgroups and predictors of one-year re-admission among late-middle-aged and older substance abuse patients. J Stud Alcohol.

[26] Moos RH, Mertens JR, Brennan PL (1994). Rates and predictors of four-year readmission among late-middle-aged and older substance abuse patients. J Stud Alcohol.

[27] Swindle RW, Phibbs CS, Paradise MJ, Recine BP, Moos RH (1995). Inpatient treatment for substance abuse patients with psychiatric disorders: a national study of determinants of readmission. J Subst Abus.

[28] Sytema S, Burgess P (1999). Continuity of care and readmission in two service systems: a comparative Victorian and Groningen case-register study. Acta Psychiatr Scand.

[29] M. M. Yamada, M. Korman, and C. W. Hughes, “Predicting Rehospitalization of Persons with Severe Mental Illness.,” *J. Rehabil.*, vol. 66, no. 2, 2000.

[30] Sullivan G, Wells KB, Morgenstern H, Leake B (1995). Identifying modifiable risk factors for rehospitalization: a case-control study of seriously mentally ill persons in Mississippi. Am J Psychiatry.

[31] Morrow-Howell NL, Proctor EK, Blinne WR, Rubin EH, Saunders JA, Rozario PA (2006). Post-acute dispositions of older adults hospitalized for depression. Aging Ment Health.

[32] C. Ng, H. Loh, and H. Yee, “The Prevalence and Associated Factors of Psychiatric Early Readmission in a Teaching Hospital, Malaysia,” *Malays. J. Psychiatry*, vol. 21, no. 1, 2012.

[33] Silva NC, Bassani DG, Palazzo LS (2009). A case-control study of factors associated with multiple psychiatric readmissions. Psychiatr Serv.

[34] Craig TJ, Bracken J (1995). A case-control study of rapid readmission in a state hospital population. Ann Clin Psychiatry.

[35] Craig TJ, Fennig S, Tanenberg-Karant M, Bromet EJ (2000). Rapid versus delayed readmission in first-admission psychosis: quality indicators for managed care?. Ann Clin Psychiatry.

[36] Goodpastor WA, Hare BK (1991). Factors associated with multiple readmissions to an urban public psychiatric hospital. Hosp Community Psychiatry.

[37] Suzuki Y, Yasumura S, Fukao A, Otani K (2003). Associated factors of rehospitalization among schizophrenic patients. Psychiatry Clin Neurosci.

[38] Nelson EA, Maruish ME, Axler JL (2000). Effects of discharge planning and compliance with outpatient appointments on readmission rates. Psychiatr Serv.

[39] Walker R, Minor-Schork D, Bloch R, Esinhart J (1996). High risk factors for rehospitalization within six months. Psychiatr Q.

[40] Kent S, Yellowlees P (1994). Psychiatric and social reasons for frequent rehospitalization. Hosp. Community Psychiatry.

[41] Parker G, Hadzi-Pavlovic D (1995). The capacity of a measure of disability (the LSP) to predict hospital readmission in those with schizophrenia. Psychol Med.

[42] Thornicroft G, Gooch C, Dayson D (1992). The TAPS project. 17: readmission to hospital for long term psychiatric patients after discharge to the community. BMJ.

[43] Touch Mercer G, Molinari V, Kunik ME, Orengo CA, Snow L, Rezabek P (1999). Rehospitalization of older psychiatric inpatients: an investigation of predictors. The Gerontologist.

[44] Mesch GS, Fishman G (1994). First readmission of the mentally ill: an event history analysis. Soc Sci Res.

[45] Priebe S (2009). Patients’ views and readmissions 1 year after involuntary hospitalisation. Br J Psychiatry.

[46] Irmiter C, Barry KL, Cohen K, Blow FC (2009). Sixteen-year predictors of substance use disorder diagnoses for patients with mental health disorders. Subst Abuse.

[47] Riordan S, Haque S, Humphreys M (2006). Possible predictors of outcome for conditionally discharged patients--a preliminary study. Med Sci Law.

[48] Klinkenberg WD (1995). Using client characteristics and treatment system variables to predict receipt of aftercare and psychiatric rehospitalization.

[49] Callaly T, Hyland M, Trauer T, Dodd S, Berk M (2010). Readmission to an acute psychiatric unit within 28 days of discharge: identifying those at risk. Aust Health Rev.

[50] Callaly T, Trauer T, Hyland M, Coombs T, Berk M (2011). An examination of risk factors for readmission to acute adult mental health services within 28 days of discharge in the Australian setting. Australas Psychiatry Bull R Aust N Z Coll Psychiatr.

[51] Nielsen B, Moltke A, Larsen JK, Grinsted P (2008). Fewer readmissions of schizophrenic patients who have contact with their own GP. Ugeskr Laeger.

[52] Owen C, Rutherford V, Jones M, Tennant C, Smallman A (1997). Psychiatric rehospitalization following hospital discharge. Community Ment Health J.

[53] Sharifi V, Tehranidoost M, Yunesian M, Amini H, Mohammadi M, Jalali Roudsari M (2012). Effectiveness of a low-intensity home-based aftercare for patients with severe mental disorders: a 12-month randomized controlled study. Community Ment Health J.

[54] Gillis LS, Koch A, Joyi M (1990). The value and cost-effectiveness of a home-visiting programme for psychiatric patients. S Afr Med J.

[55] Barker E (1996). The effect of psychiatric home nurse follow-up on readmission rates of the mentally ill.

[56] Thompson EE, Neighbors HW, Munday C, Trierweiler S (2003). Length of stay, referral to aftercare, and Rehospitalization among psychiatric inpatients. Psychiatr Serv.

[57] K. N. Zeff, S. C. Armstrong, and R. A. Folen, “Characteristics Associated With Psychiatric Readmission,” *Hosp. Community Psychiatry*, vol. 41, no. 1, 1990. 10.1176/ps.41.1.91 2153096

[58] Korkeila JA, Karlsson H, Kujari H (1995). Factors predicting readmissions in personality disorders and other nonpsychotic illness: a retrospective study on 64 first-ever admissions to the psychiatric Clinic of Turku, Finland. Acta Psychiatr Scand.

[59] Craig TJ (1997). Diagnosis, treatment, and six-month outcome status in first-admission psychosis. Ann Clin Psychiatry.

[60] Yeaman C, Gambach J, Bach B, Manker J, Diwan S, Corrigan P (2003). What happens to people receiving inpatient psychiatric services in mixed rural and urban communities?. Admin Pol Ment Health.

[61] Pfeiffer PN, Ganoczy D, Zivin K, McCarthy JF, Valenstein M, Blow FC (2012). Outpatient follow-up after psychiatric hospitalization for depression and later readmission and treatment adequacy. Psychiatr Serv.

[62] Schoenbaum SC, Cookson D, Stelovich S (1995). Postdischarge follow-up of psychiatric inpatients and readmission in an HMO setting. Psychiatr Serv.

[63] Cougnard A (2006). Pattern of health service utilization and predictors of readmission after a first admission for psychosis: a 2-year follow-up study. Acta Psychiatr Scand.

[64] Oiesvold T (2000). Predictors for readmission risk of new patients: the Nordic comparative study on Sectorized Psychiatry. Acta Psychiatr Scand.

[65] D’Ercole A, Struening E, Curtis JL, Millman EJ, Morris A (1997). Effects of diagnosis, demographic characteristics, and case management on rehospitalization. Psychiatr Serv.

[66] Kolbasovsky A (2009). Reducing 30-day inpatient psychiatric recidivism and associated costs through intensive case management. Prof Case Manag.

[67] Downing A, Hatfield B (1999). The care Programme approach: dimensions of evaluation. Br J Soc Work.

[68] Eldon Taylor C, Lopiccolo CJ, Eisdorfer C, Clemence C (2005). Best practices: reducing rehospitalization with telephonic targeted care management in a managed health care plan. Psychiatr Serv.

[69] Kuno E, Rothbard AB, Sands RG (1999). Service components of case management which reduce inpatient care use for persons with serious mental illness. Community Ment Health J.

[70] Curtis JL, Millman EJ, Struening E, D’Ercole A (1992). Effect of case management on rehospitalization and utilization of ambulatory care services. Hosp. Community Psychiatry.

[71] Rossler W, Loffler W, Fatkenheuer B, Riecher-Rossler A (1992). Does case management reduce the rehospitalization rate?. Acta Psychiatr Scand.

[72] Rossler W, Loffler W, Fatkenheuer B, Riecher-Rossler A (1995). Case management for schizophrenic patients at risk for rehospitalization: a case control study. Eur Arch Psychiatry Clin Neurosci.

[73] Dharwadkar N (1994). Effectiveness of an assertive outreach community treatment program. Aust. N. Z. J. Psychiatry.

[74] Frank D, Perry JC, Kean D, Sigman M, Geagea K (2005). Effects of compulsory treatment orders on time to hospital readmission. Psychiatr Serv.

[75] Vaughan K, McConaghy N, Wolf C, Myhr C, Black T (2000). Community treatment orders: relationship to clinical care, medication compliance, behavioural disturbance and readmission. Aust. N. Z. J. Psychiatry.

[76] Segal SP, Burgess PM (2008). Use of community treatment orders to prevent psychiatric hospitalization. Aust. N. Z. J. Psychiatry.

[77] Swartz MS, Swanson JW, Hiday VA, Wagner HR, Burns BJ, Borum R (2001). A randomized controlled trial of outpatient commitment in North Carolina. Psychiatr Serv.

[78] Maughan D, Molodynski A, Rugkåsa J, Burns T (2014). A systematic review of the effect of community treatment orders on service use. Soc Psychiatry Psychiatr Epidemiol.

[79] Haggerty JL, Reid RJ, Freeman GK, Starfield BH, Adair CE, McKendry R (2003). Continuity of care: a multidisciplinary review. BMJ.

[80] Juven-Wetzler A, Bar-Ziv D, Cwikel-Hamzany S, Abudy A, Peri N, Zohar J (2012). A pilot study of the ‘continuation of care’ model in ‘revolving-door’ patients. Eur Psychiatry.

[81] Kikuchi H, Abo M, Kumakura E, Kubota N, Nagano M (2013). Efficacy of continuous follow-up for preventing the involuntary readmission of psychiatric patients in japan: a retrospective cohort study. Int J Soc Psychiatry.

[82] Schmidt-Kraepelin C, Janssen B, Gaebel W (2009). Prevention of rehospitalization in schizophrenia: results of an integrated care project in Germany. Eur Arch Psychiatry Clin Neurosci.

[83] Komatsu H (2013). Effectiveness of information technology aided relapse prevention Programme in schizophrenia excluding the effect of user adherence: a randomized controlled trial. Schizophr Res.

[84] Frazier RS, Amigone DK, Sullivan JP (1997). Using continuous quality improvement strategies to reduce repeated admissions for inpatient psychiatric treatment. J Healthc Qual.

[85] Frazier RS, Casper ES (1998). Best practices: a comparative study of clinical events as triggers for psychiatric readmission of multiple recidivists. Psychiatr Serv.

[86] Zhang J, Harvey C, Andrew C (2011). Factors associated with length of stay and the risk of readmission in an acute psychiatric inpatient facility: a retrospective study. Aust N Z J Psychiatry.

[87] Prince JD (2006). Practices preventing Rehospitalization of individuals with schizophrenia. J Nerv Ment Dis.

[88] O. Ben-Arie, A. Koch, M. Welman, and A. F. Teggin, “The effect of research on readmission to a psychiatric hospital,” *Br. J. Psychiatry*, vol. 156, no. Journal Article, pp. 37–39, 1990. 10.1192/bjp.156.1.37 2297618

[89] Stahler GJ, Mennis J, Cotlar R, Baron DA (2009). The influence of neighborhood environment on treatment continuity and rehospitalization in dually diagnosed patients discharged for acute inpatient care. Am J Psychiatry.

[90] Loch AA (2012). Stigma and higher rates of psychiatric re-hospitalization: São Paulo public mental health system. Rev Bras Psiquiatr.

[91] Sledge WH, Lawless M, Sells D, Wieland M, O’Connell MJ, Davidson L (2011). Effectiveness of peer support in reducing readmissions of persons with multiple psychiatric hospitalizations. Psychiatr Serv.

[92] de Jong MH (2016). Interventions to reduce compulsory psychiatric admissions: a systematic review and meta-analysis. JAMA Psychiatry.

[93] Omer S, Priebe S, Giacco D (2015). Continuity across inpatient and outpatient mental health care or specialisation of teams? A systematic review. Eur. Psychiatry.

[94] Burns T (2013). Community treatment orders for patients with psychosis (OCTET): a randomised controlled trial. Lancet.

