# Supplementary material for: Overview of post-discharge predictors for psychiatric re-hospitalisations: a systematic review of the literature
Source: BMC Psychiatry. 2017 Jun 24;17:227. doi: 10.1186/s12888-017-1386-z (PMC5483311; doi:10.1186/s12888-017-1386-z)
Supplement: Supplementary file 1 — Detailed search strategies (includes the specific search strategies used to identify relevant studies for the CEPHOS-LINK systematic review on post-discharge factors and psychiatric readmission) (PDF 82 kb) [file 12888_2017_1386_MOESM1_ESM.pdf]

Additional file 1: Detailed search strategies

1. **Ovid Medline**

Search date June 4 2014

PSYCHIATRIC DISORDERS

1. exp "mental disorders" (MeSH) or "mentally ill" or *stress, psychological/
2. ("mental disorder*" or "mental illness" or "mentally ill" or "mental disease" or "psychological problem*" or psychiatr* or "mental health problem*").ti.
3. 1 or 2

AND REHOSPITALISATION

4. "patient readmission" (MeSH)
5. exp "continuity of care" (MeSH)

6 ("rehospitali*" or "readmission" or "revolving door").ab

7. 4 or 5 or 6
8. 3 and 7

Search results:

#	**Searches**	**Results**
1	("mental disorder*" or "mental illness" or "mentally ill" or "mental disease" or psychiatr*).ti.	96314
2	exp *"mental disorders"/ or exp *"mentally ill persons"/	783358
3	1 or 2	825084
4	("rehospitali*" or "readmission" or "repeated admission" or "revolving door").ti.	2031
5	*Patient Readmission/	3193
6	4 or 5	3756
7	3 and 6	743
8	limit 7 to yr="1990 -Current"	525
9	(editorial* or letter* or news* or comment*).pt.	1505334
10	8 not 9	490

2. PsycINFO

Search date June 6 2014

PSYCHIATRIC DISORDER

TI (mental* or "mental dis*" or "problem behav*" or "behav* problem*" or psychiatr* or psychological) OR KW (mental* or "mental dis*" or "problem behav*" or "behav* problem*" or psychiatr*)

AND REHOSPITALISATION

TI(rehospitali* or readmission or "continuity of care" or "revolving door") OR KW(rehospitali* or readmission or "continuity of care" or "revolving door")

Search results:

S4 **Limiters** - Publication Year: 1990-2014 **Search modes** - Boolean/Phrase 241

S3 S1 OR S2 423

S2 ((MM "Mental Disorders" OR MM "Adjustment Disorders" OR MM "Affective Disorders" OR MM "Alexithymia" OR MM "Anxiety Disorders" OR MM "Autism" OR MM "Chronic Mental Illness" OR MM "Dementia" OR MM "Dissociative Disorders" OR MM "Eating Disorders" OR MM "Elective Mutism" OR MM "Factitious Disorders" OR MM "Gender Identity Disorder" OR MM "Hysteria" OR MM "Impulse Control Disorders" OR MM "Koro" OR MM "Mental Disorders due to General Medical Conditions" OR MM "Neurosis" OR MM "Paraphilias" OR MM "Personality Disorders" OR MM "Pervasive Developmental Disorders" OR MM "Pseudodementia" OR MM "Psychosis" OR MM "Schizoaffective Disorder") OR (MM "Psychiatric Patients")) OR (TI ("mental disorder*" or "mental* ill*" or "psychiatric disorder*" or "psychiatric patient*")) 185,653

S1 (TI(rehospitali* or readmission or "repated admission" or "repeated hospitali*" or "revolving door") OR SU("Psychiatric Hospital Readmission")) 1,263

3. ProQuest Health Management

Search date June 6 2014

PSYCHIATRIC DISORDERS

AB("mental health" or "mental dis*" or "problem behav*" or "behav* problem*" or psychiatric or psychological)

AND REHOSPITALISATION

AB("rehospitali*" or "readmission" or "continuity of care" or "revolving door")

Search results:
S4 S3 limited to 1990-2014 37 references
S3 S1 AND S2 38 references
S2 (MJMESH.EXACT.EXPLODE("Patient Readmission:E.02.760.400.620") OR MJMESH.EXACT.EXPLODE("Patient Readmission:N.02.421.585.400.620")) OR ti((rehospitali* or readmission or "repated admission" or "repeated hospitali*" or "revolving door")) 882
S1 SU.EXACT("Mental disorders") OR (MJMESH.EXACT.EXPLODE("Mental Disorders Diagnosed in Childhood") OR MJMESH.EXACT.EXPLODE("Mental Disorders")) OR ti(("mental disorder*" or "mental* ill*" or "psychiatric disorder*" or "psychiatric patient*"))

4. **OpenGrey (formely SIGLE)**
Search date June 10 2014

PSYCHIATRIC DISORDERS
psychiatry OR "psychiatric patient" OR "mental disorder" OR "mental disease" OR "behavioral disorder"

AND REHOSPITALISATION
rehospitalisation OR readmission OR "continuity of care" OR "revolving door"

Search results:
(psychiatr* OR mental*) AND (rehospitali* OR readmission OR readmitted OR “repeated admission” OR "revolving door") 3 references

5. **Google Scholar**
Search date June 4 2014

Search results:
limit to years 1990-
allintitle: rehospitalization psychiatric 49 references
allintitle: rehospitalization mental 21 references
allintitle: readmission mental 34 references
allintitle:readmission psychiatric 113 references
allintitle: "revolving door" mental 26 references
allintitle: "revolving door" psychiatric 14 references
allintitle: repeated admission mental 0 references

allintitle: repeated admission psychiatric 0 references
