# Supplementary material for: Overview of post-discharge predictors for psychiatric re-hospitalisations: a systematic review of the literature
Source: BMC Psychiatry. 2017 Jun 24;17:227. doi: 10.1186/s12888-017-1386-z (PMC5483311; doi:10.1186/s12888-017-1386-z)
Supplement: Supplementary file 2 — Evaluation table (study description table which includes: author, year and country of each publication, the diagnostic category for the study population, the study design, the aim of the study, the recruitment and follow up interval, the post discharge factors investigated by the study, the pre-discharge factors investigated and whether the factor was found to be significant) (PDF 200 kb) [file 12888_2017_1386_MOESM2_ESM.pdf]

Additional file 2: Evaluation table

Author and year	Country	Study population	Diagnosis category	Study design	Aim of the study	Recruitment interval	Follow-up interval	PD factor investigated	Factor is significant (univariate OR multivariate)
Barker et al. 1999	USA	68 patients admitted to the mental health unit during a 30-day period	F3	Prospective cohort	To evaluate whether hospital readmission rates of patients with depression decreased as a result of psychiatric nurse home visits.	30 days	60 days	Psychiatric nurse home follow-up : The nurse came to the patients home to deliver mental health interventions (assessment of stressors, assessment of coping mechanisms and teaching new effective coping skills, teaching about the therapeutic regimen, and reinforcing compliance) and to provide close follow-up; three times a week for 9 weeks.	Yes
Becker et al.2007	USA	Voluntary admitted adult patients with schizophrenia, with LOS between 1 and 365	F2	Prospective cohort LADB	To investigate voluntary readmission and possible risk factors	10 years	5 years	Follow-up within 30 days	Yes
Ben-Arie et al. 1990	South Africa	Patients with illness severe enough to warrant admission to the general wards of a large psychiatric hospital in Cape Town (Valkenberg Hospital).	No limitation	Case control	To assess whether and in what way research procedures may have affected outcome in a large study designed to assess the course of major psychiatric disorder in a cohort of patients.	8 1/2 months	2 years	Being part of a research program: Experimental group patients were intensively assessed on index admission by way of interviews with patients and relatives, and 4 six monthly home visits by psychiatric nurses.	Yes
Bernet 2013	USA	Veterans at High Risk of Suicide (N=124)	No limitation	Prospective cohort LADB,	To determine a relationship between the number of mental health contacts in the thirty days after discharge, and the likelihood of psychiatric readmission in the 12 months after the initial hospitalization for a psychiatric diagnosis.	17 months	12 months	Follow-up within 30 days	Yes
Boden et al. 2011	Sweden	Patients with a first hospitalization for schizophrenia or schizoaffective disorder between	F2	Prospective cohort, Record linkage study	To identify risk factors for re-hospitalization of patients with recent onset schizophrenia or schizoaffective disorder in a	1 year	Variable (max 2 years)	Early post-discharge non-adherence to antipsychotics : not having filled a prescription of antipsychotic medication within the first week after discharge from the index hospitalization	Yes

		2006 and 2007 (n = 861)			population-based cohort study.				
Browne et al. 2013	Australia	People with schizophrenia discharged to two types of accommodation. The types of accommodation: own home and for-profit boarding house (N=391).	F2	Retrospective cohort LADB,	To investigate the relationship between the type of housing people with schizophrenia are discharged to, their re-admission rates and length of stay in hospital, using archival data	3 years and 6 months	NA	Type of housing : persons own home versus for-profit boarding house	Yes
Burgess et al. 2006	Australia	All 128,893 discharges from inpatient care	No limitation	Prospective cohort, LADB	To test the hypothesis that hospital discharges made subject to CTOs are associated with a reduced risk of readmission.	9 years	Variable	Community treatment orders (CTO) : CTO is defined as = a form of involuntary outpatient treatment (IOP) used principally for conditional discharge from hospital	Yes
Callaly et al. 2010	Australia	Patients discharged during the 2005-2006 financial year and who have been readmitted within 28 days vs. Patients who were not readmitted	No limitation	Case control	To examine factors that could help identify those most at risk of readmission to an acute psychiatric in-patient unit within 28 days of a discharge.	1 year	28 days	Days from discharge to community follow-up : first contact with the CMHT following discharge	Yes u
Callaly et al. 2011	Australia	Patients discharged from eight Australian adult acute mental health inpatient services (N=475)	No limitation	Case control	To identify risk factors associated with readmission within 28 days of discharge	1 year	28 days	Follow up by GP : discharge plan sent to GP	Yes
CG et al. 2012		Psychiatric patients discharged from a teaching hospital in Malaysia (N=202)	No limitation	Prospective cohort	To determine the early readmission rate among the psychiatric patients discharged from a teaching hospital in Malaysia and the he associated factors	8 1/2 months	6 months	Compliance to treatment : defined as missing medication as prescribed for 2 consecutive weeks or more	Yes
Claassen et al. 2005	USA	75,815 patient visits made to a hospital-based psychiatric emergency service for mental health care between January 1, 1995, and December 31, 2002	No limitation	Natural historic experiment LADB,	This study examined whether implementation of managed care in a public mental health system affected return visits to psychiatric emergency services within 180 days of an index visit	7 years	6 months	Managed care : system intervention	Yes

Cougnard et al.2006	France	First-admitted subjects with psychosis (n = 84)	F2	Prospective cohort	To explore the pattern of health service utilization over 2 years following a first admission for psychosis and the baseline characteristics predicting readmission	1-year	2 years	Number of contacts with mental health providers : Medical professionals (Public psychiatrist, Private psychiatrist, Specialist (other than psychiatrist), GP) and Other health professionals (Community psychiatric nurse, Psychologist, Psychotherapist, Psychomotrician) and Mental health professionals (Social worker, other)	No
Craig et al. 2000	USA	First-admission patients diagnosed with schizophrenia, bipolar disorder with psychosis, and major depression with psychosis (N=674 at baseline, 603 at 6 month follow-up)	F2	Prospective cohort	Examined clinical correlates of rapid readmission to a psychiatric inpatient service (less than 3 months after discharge) compared to delayed readmissions (3-12 months) in first-admission patients diagnosed with schizophrenia, bipolar disorder with psychosis, and major depression with psychosis	6 years	6 month and 12 month	Locus of care : Defined as locus of follow-up care	No
Craig et al. 1995	USA	All patients readmitted to a large state psychiatric centre during a 4 month period (Sept.-Dec. 1990), having been discharged from the same hospital less than 90 days prior to readmission.	F2	Case control	To identify clinical and service system characteristics that might differentiate rapidly readmitted psychiatric inpatients from matched samples of patients readmitted after a community tenure of more than 90 days and patients discharged with no readmission within a 6-month period.	4 months	3 months	Type of housing : Discharged home, supported community residences or proprietary, nursing, or boarding homes, other	No
Craig et al. 1997	USA	202 first-admission inpatients with DSM-III-R schizophrenia spectrum (N = 96), psychotic bipolar disorder (N = 64), and psychotic depression (N = 42 202 first-admission inpatients with DSM-III-R schizophrenia spectrum (N = 96), psychotic bipolar	F2	Prospective cohort	To investigate the 6-month clinical and psycho-social outcomes for patients diagnosed with a psychotic illness and the association among clinical and psycho-social outcome variables as well as treatment modalities	A 3 years	6 months	Continuity of treatment : rated as continuous if the patient reported having regularly participated in some form of treatment during the 6-month follow-up period vs. Brief or no treatment	Yes

		disorder (N = 64), and psychotic depression (N = 42)							
Curtis et al. 1992	USA	Number of medication (only) visits during the 6-month period (a subset of 4; range = 0-22 medication-only visits).	F1, F2	Case control	To explore in a case-control study whether intensive outreach case management would reduce rates of psychiatric rehospitalisation after an index hospitalization and increase patients use of outpatient ambulatory care services.	18 months	Different time periods (35 to 52 months)	Case management : Multidisciplinary intensive outreach case management (IOCM) or less intensive community support system (CSS) case management services compared with routine aftercare (RA) but no case management	Yes
D'Ercole et al. 1997	USA	Inpatients discharged from Harlem Hospital Centre in 1984-1985 (N=189)	F2	RCT	To assess the effects of case management and patients' characteristics on the use of inpatient psychiatric services.	18 months	12 months	Case management: The case management team implemented the discharge treatment plan prescribed for each patient assigned to the case management group and monitored the patient's health problems, psychopathology, family and housing problems, and use of social services.	Yes
Dharwadkar 1994	Australia	50 patients with the most re-admissions to the Department of Psychiatry over a 12 month period (1989-90) and a primary diagnosis of schizophrenia or major affective disorder	F2, F3	Natural historic experiment	To outline the effects upon admission rates and time in hospital, after implementation of a Adult Community Team program, for 50 of the most disturbed patients attending our service with a diagnosis of a psychotic disorder.	12 months	1 year after the program	Adult community treatment program: The ACT program incorporates a broad spectrum of services to patients who have chronic psychiatric conditions and who are especially prone to relapse. This is achieved by active and co-ordinated case management and intensive psychiatric follow-up. The program offers home-based treatment and support to clients and their families. It facilitates the integration of clients into supportive community based networks	Yes
Downing et al. 1999	UK	Patients with severe and enduring mental disorders discharged from the hospital psychiatric unit (N=35)	F2	Natural historic experiment	To evaluate CPA (Care Programme Approach)	3 months	6 months	Case management: The Care Programme Approach (CPA): included such features as a keyworker/case manager offering a continuous relationship and coordinated care, assessment and intervention over a range of 'needs', multidisciplinary working in the community, and involvement of user and carer.	No
Eldon Taylor et al. 2005	USA	60 clients with multiple inpatient admissions	F1, F2, F3	Natural historic experiment	To formally evaluate the effectiveness of the telephonic targeted care management program.	12	12	Case management: The major difference from traditional case management programs is that the program employed telephonic interventions without a face-to-face component.	Yes

Frank et al. 2005	Canada	42 psychotic patients who received compulsory community treatment orders	F0, F1, F2, F3	Prospective cohort	To evaluate the effect of compulsory community treatment orders on subsequent time out of the hospital.	2 years	2 years	Community treatment orders (CTO)	Yes
Frazier et al. 1998	USA	Consumers who had three or more inpatient admissions to a network hospital in the South-eastern Area during the 18-month index period (N=158)	No limitation	Prospective cohort	To assess the effectiveness of TIPS	18 months	4 years	Relapse prevention program : Triggers Intervention and Prevention System (TIPS)	Yes
Frazier et al. 1997	USA	88 consumers who had had more than two hospitalizations of any length within a 12-month period.	No limitation	Prospective cohort	To assess the effectiveness of TIPS	18 months	2 years	Relapse prevention program : TIPS	Yes
Gillis et al. 1990	South Africa	All patients admitted to hospital for 3 consecutive months	No limitation	Case control	To investigate the effectiveness of home visits in reducing readmission rates	3 months	1 year	Home visits : Not clear by whom	Yes
Goodpastor et al. 1991	USA	207 Patients with frequent re-admissions (three or more times)	No limitation	Prospective cohort	Studied factors associated with multiple readmissions	2 years	NA	Compliance to treatment : Non-compliance to medication	Yes
Grinshpoon et al. 2011	Israel	Patients discharged from the Tirat Carmel psychiatric hospital in Israel (N=908)	F0, F2, F3, F4, F6	Prospective cohort, Record linkage study	To examine a) the association between continuing care and time to rehospitalisation; and b) the predictors of time to first outpatient contact after discharge from psychiatric hospital.	1 year	180 days	Visits to OP after index discharge: making or not an outpatient visit within 180 days of key discharge.	Yes
Hassan et al. 2009	USA	Patients age 18-64 years who were discharged from a hospital with a diagnosis of bipolar disorder and given a prescription for an antipsychotic 0-14 days after discharge comprised the study sample. (N=1973)	F3	LADB, Retrospective cohort	To study the relationship between nonadherence to antipsychotic medication after hospital discharge and risk of rehospitalisation in patients who were previously hospitalized for treatment of bipolar disorder.	5 and a half years	NA	Compliance to treatment : Medication possession ratio (MPR); The MPR was calculated as the number of unique days any antipsychotic medication was prescribed to a patient during the 365 days after medication initiation divided by the total number of days in the post index period	Yes
Ilgen et al. 2008	USA	All patients with co-occurring substance	No limitation	Prospective cohort	The association between continuing outpatient care	1 year	90 days	Continuity of care: received continuing outpatient psychiatric care in the 30-day	Yes

		use and psychiatric disorders discharged from an inpatient psychiatric setting in the Department of Veterans Affairs (VA) between July 1, 2004, and June 30, 2005 (N=26,826).		LADB	for a psychiatric disorder, a substance use disorder, or both and decreased risk of readmission to psychiatric care after an index episode of inpatient psychiatric treatment			period after discharge from the index episode and before any subsequent psychiatric rehospitalisation and the day on which this care occurred. This information was used to create a time-dependent indicator variable of psychiatric continuing care (coded no or yes)	
Irmiter et al. 2009	USA	Individuals admitted to inpatient psychiatry (1982 to 1987) with at least one rehospitalisation within a 16-year period (N=1350)	No limitation	Prospective cohort,	The focus of the study was on determining and comparing the characteristics and predictors of a SUD as comorbidity at index admission versus as post-discharge variable by comparison with those without SUD	20 years	16 years	Alcohol/Substance abuse : Diagnostic of a SUD at baseline vs. Post baseline	Yes
Irmiter et al. 2007	USA	Patients with SMI (schizophrenia, schizoaffective, or bipolar disorder) who were discharged in Fiscal Year 1998 (FY98) from VA inpatient psychiatric settings (N = 35,527).	F2, F3	Prospective cohort, Record linkage study	To evaluate prevalence rates, timing, and risk factors for re-institutionalization following psychiatric discharges among patients with SMI.	1 year	7 years	Type of housing : Homelessness as a risk factor	Yes
Juven-Wetzler et al. 2012	Israel	All patients who were hospitalized three times or more during the past 12 months (N=35)	No limitation	Case control	To test the efficiency of continuation of care (COC) treatment by inpatient caregivers as compared to treatment administered by outpatient services for ‘’revolving door’’ psychiatric patients	12 months	18 months	Continuity of care : continuing follow-up in the ward, by the same staff, instead of being referred to the outpatient department	Yes
Kent et al. 1994	Australia	50 patients with frequent readmissions to the South Australian Mental Health Services over 3 years	No limitation	Descriptive	To identify factors that commonly contributed to the decision to re-hospitalize patients who made heavy use of mental health services.	1 year	NA	Type of housing : problems with accommodation	NA
Kikuchi et al. 2013	Japan	All discharged psychiatric patients (N=200)	F2	Retrospective cohort	To evaluate the efficacy of a new continuous follow-up system (consisting in 4 3 monthly follow-up up	3 years	1 year	Continuity of care : continuous follow-up at 3, 6, 9 and 12 months after discharge by phone or home visits	Yes

					sessions either by telephone or by home visits)				
Kim 2011	USA	Patients who had at least two inpatient psychiatric hospitalizations (case group, N=17,852) compared with matched patients not re hospitalized in the same period of time as the case (N=35,511)	F3	LADB, case control	To assess whether increased frequency of clinical monitoring during the high-risk period of 12 weeks after discharge from a psychiatric hospitalization reduced subsequent rehospitalisation in a national cohort of Veterans Health Administration patients receiving depression treatment between 1999 and 2004.	5 years	NA	Continuity of care : Clinical monitoring; Monitoring intensity was expressed as a rate of visits (in person visits or visits by telephone) per 84 days	No
Kolbasovsky 2009	USA	All eligible health plan members discharged from an acute inpatient hospitalization with a primary psychiatric diagnosis during a 1-year time period.		Case control	To determine the impact of ICM on 30-day inpatient psychiatric recidivism and associated costs among adult health plan members at elevated risk of psychiatric hospitalization	1 year	30 days	Case management: Intensive case management. ICM services are typically provided by a social worker, nurse, or other mental health clinician who maintains the primary responsibility of keeping in contact with the patient, assessing his or her needs, helping ensure that those needs are met, providing brokerage and advocacy, conducting activities of daily living skills training, and linking patients with educational and community resources.	Yes
Komatsu et al. 2013	Japan	Outpatients with schizophrenia	F2	RCT	To evaluate the effectiveness of the ITAREPS excluding the effect of user adherence to the protocol of the program (N=45).	NA	12	Relapse prevention program: Information Technology Aided Relapse Prevention Programme in Schizophrenia (ITAREPS). The ITAREPS presents a mobile phone-based telemedicine solution for weekly remote patient monitoring and disease management in schizophrenia and psychotic disorders in general. The program provides health professionals with home tele monitoring via a PC-to-phone short message service (SMS) platform that identifies prodromal symptoms of relapse, to enable early intervention and prevent unnecessary hospitalizations.	Yes
Korkeila et al. 1995	Finland	All first-ever episodes of inpatient care, excluding psychotic	No limitation	Prospective cohort	To study factors predicting readmissions and follow-up	2 years	5 years	Locus of care: The possible follow-up treatment setting was grouped as 1) specialized psychiatric treatment, 2)	No

		and organic mental disorders, during a 2-yr period in a Finnish psychiatric clinic (N=64).			treatment of all first-ever episodes of inpatient care.			basic health care, 3) private therapist or psychiatrist or 4) polyclinic follow-up treatment by a nurse familiar to the patient.	
Kuno et al. 1999	USA	Medicaid clients, ages 18 to 59, with a diagnosis of schizophrenia or chronic mood disorder, and a psychiatric hospitalization for at least 60 days within a year in community hospitals in Philadelphia between November 1, 1988 and December 31, 1991. (N=164)	F2, F3	Case control	To compare 2 types of case management: case management (CM) which provided the service coordination functions, and Intensive Case Management (ICM) which consisted of both the coordination function and the provision of direct support to the client.	3 years	1 year	Case management: Intensive Case Management (ICM) as compared with case management	Yes
Loch 2012	Brazil	One hundred-sixty-nine individuals with bipolar and psychotic disorder in need of hospitalization in the public mental health system.	F2, F3	Prospective cohort	To assess re-hospitalization rates of individuals with psychosis and bipolar disorder and to study determinants of readmission.	4 months	1, 2, 6, 12 months	Visits to OP after index discharge : not attending outpatient consultations as a risk factor	Yes
Mark et al. 2013	USA	1375 individual records of patients with a baseline admission between 1982 and 1987 and re-hospitalized at least once over the next 16 years.	No limitation	Prospective Cohort, LADB	To provide data on readmissions for M/SUDs to inform debate over hospital readmission as an actionable quality performance indicator.	6 years	30 days (days 8-30)	Receipt of medication : the percentage receiving a prescription fill for M/SUD,	Yes
Mesch et al. 1994	Israel	First-time patients with schizophrenia admitted to a state mental hospital in Israel (494).	F2	Prospective cohort	To examine the effects of living arrangements following 1st release, work placement after discharge, and length of hospitalization on the probability of readmission (RA) of 494 Ss (aged 22-54 years) admitted to a state mental hospital in Israel	2 years	5 years	Type of housing : Living arrangements (family of orientation, family of procreation, living alone)	Yes

Moos et al. 1994	USA	Inpatients with only an alcohol or drug dependence diagnosis (n = 11,652); inpatients with an alcohol or drug psychosis (n = 3,510); and inpatients with an alcohol or drug disorder and a concomitant psychiatric disorder (n = 5,977).	F1	Prospective cohort, Record linkage study	To examine treatment utilization and 1-year re-admission rates among three diagnostic subgroups of late-middle-aged and older substance abuse inpatients in Department of Veterans Affairs (VA) Medical Centres.	1 year	1 year	Visits to OP after index discharge: Whether patients obtained outpatient mental health or medical care during this interval, and, for those who did, the number of visits for each type of care.	No
Moos et al. 1994	USA	Late-middle-aged and older (age 55+) substance abuse inpatients (N = 16,066)	F1	Prospective cohort,Record linkage study	To examine treatment, diagnoses and readmission among late-middle-aged and older (age 55+) substance abuse inpatients (N = 16,066) in Department of Veterans Affairs Medical Centres.	1 year	4 years	Visits to OP after index discharge: Includes the percentage of patients who obtained outpatient mental health or medical care in the 4 years after the index episode, and the number of visits for each type of care.	Yes
Moos et al. 1995	USA	33,323 substance abuse patients discharged from 88 Department of Veterans Affairs (VA) substance abuse treatment programs in fiscal year 1991.	F1	Prospective cohort,LADB	To explore the associations between specific program characteristics intended to foster patient improvement and case mix-adjusted rates of readmission for inpatient substance abuse or psychiatric care.	1 year	1 year	Visits to OP after index discharge: post discharge outpatient mental health care was examined for the 1-month interval after the index episode of inpatient care	Yes
Morrow-Howell et al. 2006	USA	199 for older adults hospitalized for depression and discharged to the community.	F3	Prospective cohort	To analyse factors associated with six-month post-acute dispositions (continuous community stay, medical hospitalization, psychiatric rehospitalisation, nursing home placement, death) for older adults hospitalized for depression and discharged to the community.	2 years	6 months	Compliance to treatment : Medical compliance as judged by family	Yes
Nelson et al. 2000	USA	3,113 patients discharged from inpatient psychiatric care in 1998	No limitation	Prospective cohort	To examine whether patients discharged from inpatient psychiatric care would have lower rehospitalisation rates if they kept an outpatient follow-up appointment after discharge	1 year	90, 180, 270, and 365 days	Compliance to appointments : complied with at least an appointments vs. Did not comply to any	Yes

Nielsen et al. 2008	Denmark	96 schizophrenic patients discharged	F2	Prospective cohort	To investigate to which extent the GP is part of the follow-up treatment of schizophrenic patients after their discharge from a psychiatric department.	1 year	12 months	Follow up by GP : Contact with the GP after discharge	Yes
Niksalehi et al. 2011	Iran	62 patients with schizophrenia discharged from hospital	F2	Experimental	To compare the impact of two follow up methods in rate of rehospitalisation, length of hospitalization and mental condition of schizophrenics.	NA	4 months	Home visits	Yes
Oiesvold et al. 2000	Norway, Sweden, Finland, Denmark	837 consecutive 'new' patients admitted to psychiatric hospitals in four Nordic countries.	No limitation	Retrospective cohort	To identify predictors for readmission risk.	1 year	1 year	Visits to OP after index discharge : receipt of aftercare	Yes
Owen et al.1997	Australia	128 inpatients (aged 16.7-80.6 years)	No limitation	Prospective cohort	To examine the relationship between rehospitalisation and the nature of psychiatric aftercare in a well-integrated hospital and community based psychiatric service.	6 months	6 months	Follow up by GP : time in hours	Yes
Parker et al. 1995	Australia	118 subjects with an admission diagnosis of schizophrenia	F2	Prospective cohort	Test the capacity of Life Skills Profile (LSP) to predict hospital readmission in those with schizophrenia	6 months	1 year	Type of housing : alone, with family/friends, Boarding house, hostel	Yes u
Pfeiffer et al. 2012	USA	56,785 Veterans Health Administration patients with an inpatient stay for major depression between 2005 and 2010.	F3	Prospective cohort	To assess whether timely post discharge follow-up, a health system quality indicator, corresponded with improved longer-term post hospital care for depression	5 years	NA	Follow-up within 7 days	No
Postrado et al. 1995	USA	559 patients with severe mental illness	SMI	Prospective cohort	To examine whether rehospitalisation of patients with severe and persistent mental illness could be predicted by patients' quality of life.	4 years	10 months	Quality of life : Satisfaction with family relations	Yes
Priebe et al. 2009	UK	1570 consecutive patients between 18 and 65 years, , admitted under Sections 2, 3 and 4 of	No limitation	Prospective cohort	To assess involuntary readmissions and patients' retrospective views of the justification of the admission as 1-year outcomes and to	2 years	1 year	Type of housing : living alone vs living with others	Yes

		the Mental Health Act 1983			identify factors associated with these outcomes				
Prince 2006	USA	264 persons with schizophrenia	F2	Prospective cohort	To examine the extent to which inpatient readmission among 264 persons with schizophrenia was averted by interventions addressing medication education, symptom education, service continuity, social skills, daily living, daily structure, and family issues.	1 and a half years	3 months	Other interventions : interventions addressing medication education, symptom education, service continuity, social skills, daily living, daily structure, and family issues	Yes
Riordan et al. 2006	UK	75 individuals conditionally discharged in the West Midlands between 1 April 1987 and 1 April 2000	No limitation	Prospective cohort	To identify variables among a cohort of conditionally discharged patients in the West Midlands that would predict whether an individual was more likely to be readmitted to hospital, involved in a serious incident, to be recalled to hospital or given an absolute discharge.	3 years	13 years	Alcohol/Substance abuse	Yes
Rossler et al. 1992	Germany	162 patients dismissed from psychiatric hospitals	No limitation	Case control	To estimate the effect of case management on the rehospitalization rate.	2.5 years	2.5 years	Case management	No
Rossler et al. 1995	Germany	97 schizophrenic patients in the aftercare of case management services	F2		To assess effectiveness of case management in reducing rehospitalization	2.5 years	2.5 years	Case management	No
Schmidt-Kraepelin et al. 2009	Germany	46 "high utilizing" patients with Schizophrenia	F2	Case control	To test whether patients who receive guideline-adherent complex interventions are less likely to be readmitted	6 months	12 months	Relapse prevention program	Yes
Schoenbaum et al. 1995	USA	580 psychiatric inpatients discharged from treatment in 2 divisions of a health maintenance organization.	No limitation	Prospective cohort	Examined the relationship between follow-up and rehospitalisation	1 year	1 year	Follow-up within 30 days	Yes
Sharifi et al. 2012	Iran	130 patients with schizophrenia, schizoaffective disorder or bipolar disorder	F2, F3	RCT	To examine the effectiveness of a low-intensity home-based aftercare service	NA	1 year	Home visits	Yes

Silva et al. 2009	Brazil	307 adults admitted to either of two public psychiatric hospitals in southern Brazil during a 12-month period and who had three or more psychiatric admissions in the two years before the current admission.	No limitation	Case control	To explore factors associated with multiple psychiatric admissions.	1 year	NA	Follow up by GP : Registered with a primary care unit	No
Sledge et al. 2011	USA	74 patients were 18 years or older with major mental illness and had been hospitalized three or more times in the prior 18 months (36 TAU, 36 TAU plus peer mentor)	F2, F3	RCT	To examine the feasibility and effectiveness of using peer support to reduce recurrent psychiatric hospitalizations.	2 years	9 months	Peer support	Yes
Stahler et al. 2009	USA	380 patients who were dually diagnosed with at least one mental disorder and a substance use disorder and discharged from an acute psychiatric inpatient care unit.	No limitation	Prospective cohort	To analyse neighbourhood and individual factors predicting initial outpatient treatment attendance and rehospitalization within 1 year among patients who were dually diagnosed with at least one mental disorder and a substance use disorder.	14 months	1 year	Geographical variables	Yes
Sullivan et al. 1997	USA	Individuals with schizophrenia at increased risk for rehospitalization (101 recidivists (cases) matched to 101 non recidivists (controls))	F2	Case control	Investigated the extent to which aberrant behaviours place individuals with schizophrenia at increased risk for rehospitalization.	3 months	NA	Behaviour : (1) refusing to eat, (2) not keeping self-clean or well-groomed, (3) isolating self, (4) wandering away from home or getting lost, (5) talking to people who weren't really there, (6) having a temper tantrum, (7) doing strange or bizarre things, such as dressing inappropriately, (8) acting like others were after him/her or plotting against him/her, (9) verbally or physically threatening or attacking others, and (10) talking about or attempting to harm self	Yes
Sullivan et al. 1995	USA	101 recently readmitted psychiatric Ss (with a primary	F2	Case control	To identify risk factors for rehospitalization in a seriously mentally ill	3 month	NA	Type of housing : Not living with family	Yes

		diagnosis of schizophrenia) were compared with 101 previously hospitalized community-dwelling controls			population, focusing on factors that have the potential to be modified through community-based interventions.				
Suzuki et al. 2003	Japan	67 hospitalized patients vs controls selected from the outpatients who were matched by age, gender, and the period after the last discharge (n = 62).	F2	Case control	To identify the associated factors of rehospitalization in schizophrenic patient.	4 months	NA	Compliance to treatment : medication compliance	Yes
Swartz et al. 2001	USA	331 involuntarily hospitalized patients awaiting discharge under OC	F2, F3	RCT	To provide empirical data on involuntary outpatient commitment and to evaluate its effectiveness in improving outcomes among persons with severe mental illnesses.	3 years	12 months	Community treatment orders (CTO) : outpatient commitment (OC	Yes
Swindle et al. 1995	USA	7,711 inpatients with both substance abuse and major psychiatric disorders.	No limitation	Prospective cohort,Record linkage study	To examine the patient case mix and program determinants of 6-month readmission rates and early treatment dropout for 7,711 VA inpatients with both substance abuse and major psychiatric disorders. .	1 year	180 days	Follow-up within 30 days : receiving two or more psychiatric aftercare visits within 30 days of discharge	Yes
Sytema et al. 1999	Australia, Netherlands	Patients with schizophrenia and related disorders	F2	Prospective cohort,Record linkage study	To compare service consumption, continuity of care and risk of readmission in a record linkage follow-up study of cohorts of patients with schizophrenia and related disorders in Victoria (Australia) and in Groningen (The Netherlands).	1 year	4 years	Visits to OP after index discharge : Out-patient contacts	No
Thompson et al. 2003	USA	1,481 patients (with a diagnosis of either a schizophrenic disorder, excluding schizophreniform disorder, or a mood disorder, excluding dysthymia) who had	F2, F3	Prospective cohort	To explore the interrelationship among aftercare, length of hospital stay, and rehospitalisation within six months of discharge in a sample of psychiatric inpatients.	2.5 years	6 months	Aftercare: aftercare was defined as referral to a psychiatric aftercare program, such as outpatient care, foster care, or a group home, not including a nursing home.	Yes.

		received inpatient care at a state psychiatric hospital from November 1991 to July 1994.							
Thornicroft et al. 1992	UK	357 psychiatric patients who had been in hospital for over one year, of whom 118 were "new" long stay and 239 "old" long stay patients	No limitation	Prospective cohort	O identify risk factors which increase the likelihood of readmission for long stay psychiatric patients after discharge from hospital.	5 years	5 years	Type of housing : living in a staffed vs in a non-staffed group home	Yes
Touch Mercer et al. 1999	USA	150 male, geropsychiatric inpatients (aged 59-88 years).	No limitation	Prospective cohort	To identify risk factors for geropsychiatric rehospitalisation.	2.5 years	1.5 year	Type of housing : Living at discharge (%): Home alone, Home with supervision, Personal care home, Long-term care	No
Vaughan at al. 2000	Australia	All patients (diagnosed with schizophrenia, schizophreniform disorder or schizoaffective disorder, or atypical psychosis) given CTOs (N=123) within a 4-yr period and a matched comparison group of patients (N=123)	F2	Case control	Investigated the readmission rate and the level of patient disturbance and community care associated with readmission following Community Treatment Orders (CTOs).	4 years	Variable (range = 12-60 months).	Community treatment orders (CTO)	Yes
Walker et al. 1996	USA	423 adult patients admitted to a voluntary psychiatric unit within a tertiary care general hospital in a rural section of North Carolina	No limitation	Prospective cohort	To identify risk factors associated with psychiatric rehospitalisation within six months,	1 year	6 months	Type of housing	Yes
Warren et al. 1994	Australia	133 people discharged from a public, rural, psychiatric hospital with diagnoses of mental illness.	No limitation	Prospective cohort	To test whether effective follow-up reduces readmission rates	3 years	1 year	Aftercare : 4 principles for effective follow up: focus on acute stress, medication strategy; goal oriented focus on chronic stress, ongoing and systematic	Yes
Yamada et al. 2000	USA	163 SMI patients (schizophrenia, major depression, manic	F2, F3	Prospective cohort, Record linkage study	To examine length of stay in the community of patients with SPMI and identify predictor variables.	NA	4 years	Type of housing : Patents home, boarding home, nursing home	Yes

		depressive disorder or other severe illness)							
Yeaman et al. 2003	USA	110 people who were discharged from a state hospital during a 24-month period who met criteria for recidivism, 51 individuals who were lost to follow-up, and 106 peers selected randomly as a comparison group.	F2, F3, F6	Case control	To examine three sets of hypothetical variables-demographic, diagnostic, and service-that may explain recidivism and/or lost-to-follow-up in people recently discharged from inpatient care.	2 years	NA	Contact in the community on the day of discharge : whether a community based follow-up was provided within 24 hours of discharge	No
Zeff et al. 1990	USA	246 active duty patients	No limitation	Prospective cohort	To identify factors related to the readmission of patients in the military.	6 months	3 months	Aftercare : Aftercare provider: psychiatrist vs non-psychiatrist	No
Zhang et al 2011	Australia	178 patients discharged from an acute unit	F2	Prospective cohort	To investigate predictors for the risk of readmission at an acute psychiatric inpatient unit.	1 year	1 year	Community treatment orders (CTO)	Yes
